# Minimization of Intraparenchymal Hemorrhagic Stroke Size by Optimization of Serum Lipids

**DOI:** 10.7759/cureus.4406

**Published:** 2019-04-08

**Authors:** Mark Krel, Dan E Miulli, Henry Jung, James G Wiginton, James Brazdzionis, Margaret Rose Wacker, Silvio Hoshek, Rosalinda Menoni

**Affiliations:** 1 Neurosurgery, Riverside University Health System Medical Center, Moreno Valley, USA; 2 Neurosurgery, Kaiser Permanente Los Angeles Medical Center, Los Angeles, USA; 3 Neurosurgery, Arrowhead Regional Medical Center, Colton, USA

**Keywords:** hemorrhagic stroke, cholesterol, triglycerides, hdl, ldl

## Abstract

Introduction

Intraparenchymal hemorrhages (IPHs) are the most common type of hemorrhagic stroke. One of the main associated risk factors is total cholesterol (TC) above 200. A severely decreased level of TC potentially interferes with the stabilization of the cell membrane and can potentially lead to a larger hemorrhage. Previous population-based studies have confirmed an association between low TCs and a high incidence of hemorrhagic stroke. It has been established that a TC below 200 decreases the potential for cardiovascular disease. This study suggests that the balance that needs to be achieved between these two extremes presents a unique possibility for an optimal therapeutic range of total cholesterol levels.

Materials & methods

Inclusion criteria included all adult patients with International Classification of Diseases (ICD)-9/10 code for hemorrhagic stroke, from June 2007 to June 2017. A total of 300 patients met the criteria (N=300). For each patient, the following data were collected: NIH Stroke Scale, TC level, triglyceride level, low-density lipoprotein (LDL) and high-density lipoprotein (HDL), cholesterol reducing medications, size of hemorrhage on computed tomography (CT) of the head, location of hemorrhage, and patient disposition. Statistical analysis was done using the Generalized Linear Modeling with Wald Chi-square as the statistical determinant.

Results

Intracerebral hemorrhage size is dependent on the intracranial location with brain lobes having larger bleeds. Minimum hemorrhage size was noted in TC 188-196 and this effect was statistically significant independent of location. HDL has a significant independent effect on hemorrhage size with overall minimum bleed occurring in the range of 43-51 mg/dL HDL (98-106 mg/dL for men and 43-51 mg/dL for women). This sex effect within HDL on hemorrhage size is statistically significant. There was a differential effect of HDL dependent on patient race. Asian and black patients had least IPH volume with HDL 70-79 mg/dL, while Hispanic patients had a minimum at 43-51 mg/dL. White patients required a higher HDL, 80-88 mg/dL to minimize the IPH size. The triglyceride level had a statistically significant independent effect on the bleed size with the minimum hemorrhage size occurring in the range of 205-224 mg/dL. This effect was nuanced by patient race with statistically significant minimum IPH size occurring at 144-164 mg/dL for white patients, 124-143 mg/dL for Hispanic and black patients, and 84-103 mg/dL for Asian patients. Post-hospital patient disposition was not significantly affected by any of the above predictor variables.

Conclusion

This study found TC, HDL and triglycerides in specific ranges are associated with significantly decreased hemorrhage size across all genders and hemorrhage locations. The ranges with the strongest hemorrhage-limiting effect are as follows: TC 188-196 mg/dL, HDL 43-51 mg/dL (98-106 mg/dL for men and 43-51 mg/dL for women), triglycerides 205-224 mg/dL. Lipids both below and above these ranges yield larger bleeds. It also found larger brain areas will have more extensive hemorrhage than smaller brain areas. Future work in this arena should include collaboration with cardiology to determine ideal ranges for both cardio- and neuroprotection as well as a prospective study to validate the applicability of these findings in patient care.

## Introduction

Stroke is the fifth leading cause of death in the United States. It is also the leading cause of adult disability. There are more than 150,000 stroke deaths per year, more than 795,000 new strokes, and more than 4,400,000 stroke survivors in the US with a projected 65% linear increase in these numbers through 2025 [1]. The majority of strokes fall into two categories, ischemic and hemorrhagic [2-3]. Intraparenchymal hemorrhages (IPHs) are the most common type of hemorrhagic stroke, and are associated with a higher mortality risk than ischemic strokes [3-4]. An IPH can lead to secondary effects such as perihematomal edema and can subsequently lead to an increase in intracranial pressure (ICP) [4]. IPH typically occurs in the thalamus, basal ganglia, pons, cerebellum, or cerebral lobe. There are many causes of IPH. Known direct risk factors increase the incidence of IPH occurrence in three major categories - modifiable risk factors, non-modifiable risk factors, and other factors [5].

Hypertension and smoking are some of the best described modifiable risk factors for IPH with elevated blood pressures tightly correlated with IPH incidence and amount and duration of smoking being directly proportional to IPH incidence. Non-modifiable risk factors include old age, male sex, and Asian ethnicity. Other factors include multi-parity and poor working conditions (blue-collar occupation, longer working time) [3-9]. In addition to these, one of the main associated risk factors includes a high total cholesterol level (TCL) above 200 [3, 6, 10]. Furthermore, it is established that an elevated level of low-density lipoprotein (LDL) increases the cellular oxidative stress leading to vascular endothelial dysfunction and triggering the event cascade that ultimately ends in cardio- or cerebrovascular clinical events [11].

Cholesterol must be transported to and from the cells by lipoprotein carriers since cholesterol is insoluble in the blood. TCL consists of high-density lipoprotein (HDL), LDL, triglycerides, and lipoprotein A (Lp(a)) cholesterol. HDL removes cholesterol from the blood and therefore protects against myocardial infarction. LDL transports cholesterol to the end organs and in excess throughout the blood is associated with a plaque on the walls of arteries which causes arteries to narrow and potentially clot. Triglycerides are blood-borne lipid carrier molecules. Triglycerides are a form of fat made in the liver and in higher levels are often seen with high TCLs [4]. Lp(a) cholesterol is a genetic variation of LDL and a high Lp(a) level is a known risk factor for premature development of fat deposits within arteries [2, 6, 12].

For people over the age of 18 years, TCL is considered high if it is greater than 200 mg/dL, and a TCL of less than 200 mg/dl is recommended in order to aid in the prevention of stroke and heart disease [6]. It is commonly accepted that there is a direct correlation between TCL and risk of cardiovascular and cerebrovascular disease. If TCL is higher than 200 mg/dl, or if HDL is less than 40 mg/dl in men, the risk of stroke and cardiovascular disease could increase [3, 6-7, 10, 13-16] According to the American Heart Association (AHA), a healthy level of HDL may protect against heart attack and stroke, while low levels of HDL (less than 40 mg/dl for men and less than 50 mg/dl for women) have been shown to increase the risk of heart disease [6-7, 16-19]. An elevated LDL is also a major risk factor for cardiovascular disease, and studies demonstrate that decreasing LDL helps reduce the risk of coronary heart disease [3, 6-7, 10, 13, 16-18].

Cholesterol is required for healthy cells because it stabilizes the cell membrane, thus a severely decreased level of TCL interferes with the integrity of the cell membrane [6, 10, 13-16, 20-21]. Therefore, it is possible that at some lower TCLs there is less strength of the cell membrane to counteract the force of an expanding hematoma which can potentially lead to a larger area of IPH. Previous population-based studies have confirmed an association between low TCLs and a high incidence of hemorrhagic stroke [3, 7, 10, 15-16, 20-22]. There have been no studies to date that attempt to determine an optimal range for TCL in order to prevent cardiovascular disease pathology and IPH stroke, while also avoiding interference with the ability of cholesterol to stabilize the cell membrane thereby decreasing the size of the IPH and improving patient disposition after the stroke [14, 16] Thus, if a TCL lower than 200 decreases the potential for cardiovascular disease, but an excessively low cholesterol level increases the risk of cellular membrane destabilization, and in the event of IPH, contributes to the expansion of the IPH and the clinical outcome, there exists a need to define an optimal range of goal blood lipids for patient management.

The goal of this study was to explore the relationship between the cholesterol levels, IPH size, the location of the IPH, and to find an optimal range for TCLs that will minimize the size of IPH. This data will help to guide treatment and prevention mechanisms in concert with existing data on preventing the incidence of IPH. In adding a mode for minimization of IPH size in addition to minimizing IPH incidence, we aim to improve outcomes for patients who suffer a hemorrhagic stroke.

## Materials and methods

A retrospective analysis of patient data was performed for stroke patients prospectively entered into Get with the Guidelines Stroke Registry® for the county hospital. It is the largest county by area in the United States and has a diverse range of social, economic and ethnic groups. The hospital is one of the top two busiest trauma centers and emergency departments in California, as well as a tertiary referral center for many diseases. This study was approved by the Institutional Review Board for Human Subjects.

For the purpose of this study, the Stroke Registry data were obtained for the period of Jun 2007 - December 2017 in order to obtain a list of patients by ICD9/10 code. From this list, the charts for the patients who experienced IPH were retrospectively evaluated, which yielded 300 study-eligible patients (n=300). The ages of patients ranged from 20 years through 96 years. For each patient, the following data were collected and analyzed from the electronic health record system Meditech (MEDITECH, Westwood, MA): National Institutes of Health Stroke Scale (NIHSS), TCL, LDL, HDL, cholesterol-reducing medications, size of hemorrhage on CT of the head, the location of hemorrhage, and patient disposition. Hemorrhages with a clear structural pathologic source such as tumoral hemorrhage, vascular anomalies (aneurysms, arteriovenous malformations, cavernous malformations etc.), and amyloid angiopathy were excluded from this study. The intraventricular component of the IPH was not included in the estimate of size because of the ability of blood to disperse throughout the fluid unimpeded giving a false overestimate of size.

The NIHSS is a quantifying tool used by the healthcare providers. It is an objective way to quantify the damage caused by stroke. It is composed of 11 sections that can be scored between zero and four. A score of zero indicates the normal function of that specific ability, and a score above zero indicates that damage has been done to that ability. All of the individual scores are then summed in order to calculate the patient’s total NIHSS score. The minimum possible score is a zero, and the maximum possible score is a 42. This scale is used to help determine appropriate treatment, as well as used to predict patient outcome and serve as a measure of stroke severity [23].

IPH sizes were determined using the ABC/2 model. The formula for the ABC/2 method can be explained where ‘A’ is the largest cross-sectional hemorrhage diameter by CT, ‘B’ is the largest diameter 90 degrees to ‘A’ on the same slice, and ‘C’ is the approximate number of CT slices with IPH, multiplied by the slice thickness which at our institution was uniformly 0.5 cm [24]. The product of ‘A,’ ‘B,’ and ‘C’ is then divided by two in order to approximate the volume of the bleed. Hemorrhages that overlapped in areas of the brain were classified in only the one section that contained the majority of the blood. Lesions that occurred in both the basal ganglia and thalamus were classified as thalamic lesions if the bleed was more extensive in that area relative to its total area. Similarly, hemorrhages that were in the basal ganglia and extended into a lobar region, were classified as basal ganglia bleeds if more extensive in that area relative to its size.

Since this study sought to define an optimal cholesterol range, cholesterol data were binned into discrete ranges. This same binning process was carried out for HDL, LDL and triglycerides for consistency and, again, to guide the determination of a specific optimal range. Data were then analyzed using general linear modeling and Wald Chi-square statistical analysis to determine the statistical significance. Main effects were calculated for age, race, sex, location of hemorrhage, triglycerides, TC, LDL and HDL as well as all two- and three-way interactions. All statistical analysis was done using SPSS version 23 (IBM Inc., Armonk, NY).

## Results

There were 300 patients who met inclusion criteria for the study and who had data available for each variable (age, race, sex, location of hemorrhage, triglycerides, TC, LDL and HDL). Summary descriptive statistics are presented in the table below (Table 1).

**Table 1:** Summary demographics and intraparenchymal hemorrhage frequencies by location IPH: Intraparenchymal hemorrhage

Demographics		
Sex	Count	Percentage
Male	166	55.3%
Female	134	44.7%
Age		
< 39	18	6%
40 – 49	35	11.7%
50 – 58	78	26%
59 – 68	60	20%
69 – 77	49	16.3%
78 – 87	47	15.7%
> 88	13	4.3%
Race		
Asian	15	5%
Black	44	14.7%
Hispanic	144	48%
White	93	31%
Other	4	1.3%
IPH Location		
Basal Ganglia	119	39.7%
Cerebellum	30	10%
Lobar	100	33.3%
Pons	10	3.3%
Thalamus	39	13%
Ventricle	2	0.7%

**Figure 1:**
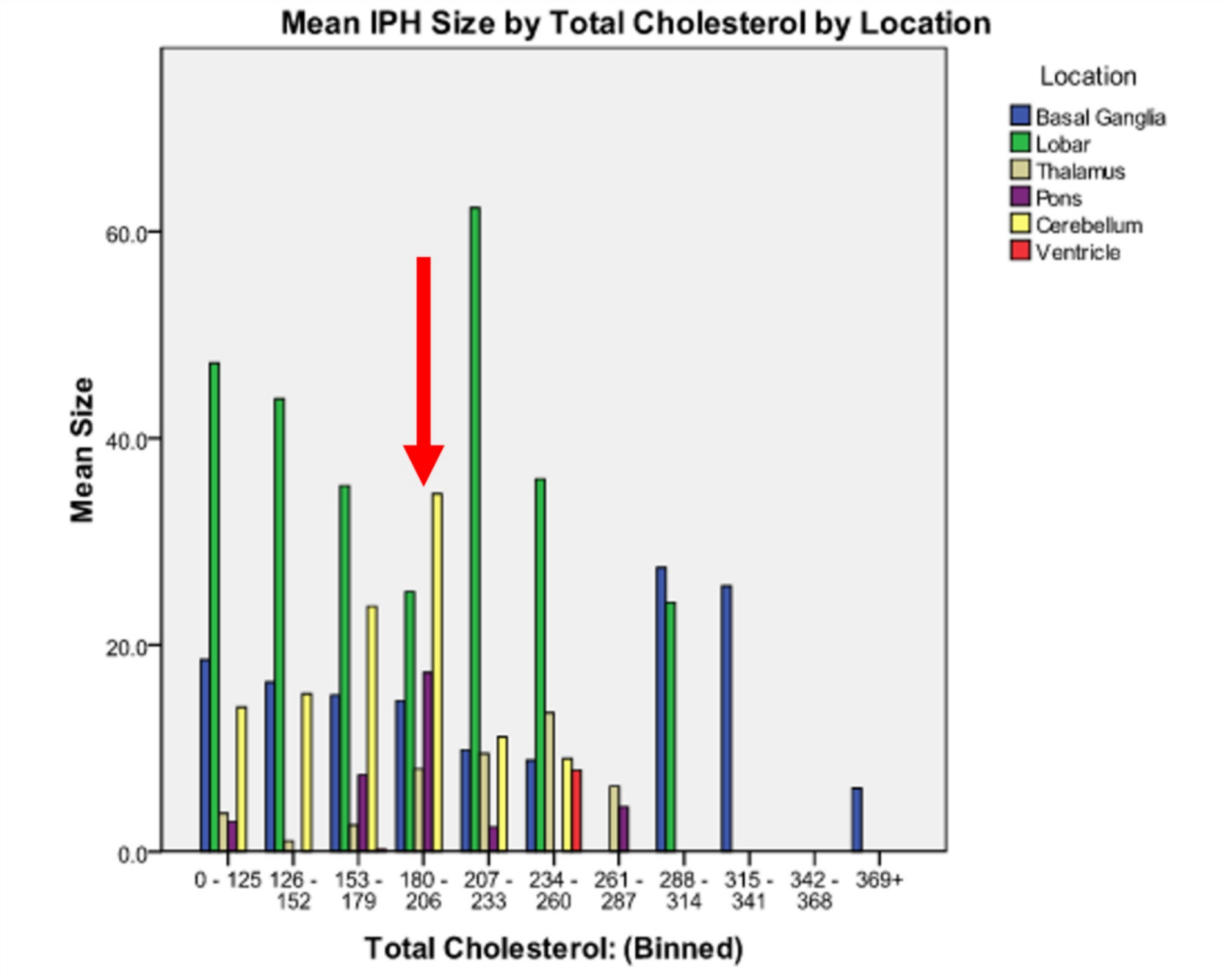
Mean intraparenchymal hemorrhage size by total cholesterol by location As expected, lobar hemorrhages are, on average, larger than other intraparenchymal hemorrhage locations. The minimum hemorrhage size occurs with total cholesterol between 188 - 196 mg/dL by calculation. In this figure, for the sake of ease of visualization since software constraints would make a graph with bins of 10 mg/dL too dense to read, the graphical bins are larger in width (24 mg/dL) than the computational bins (10 mg/dL). Patients with total cholesterol levels > 314 were very few in number and therefore the patients whose data fall above that total cholesterol level were treated as outliers for the computational analysis.

As seen in Figure 1, there exist a roughly two peak distributions of mean IPH size with a relative minimum, by calculation, occurring in the 188 -196 mg/dL TC range across all bleed locations (see arrow). This is to say that when we discount the location of the hemorrhage and strictly compare bleed sizes by TC levels, the minimum hemorrhage size occurs in the aforementioned TC range 188-196 mg/dL. Performing the analysis mathematically using a generalized linear model accounting for main effects and all factorial interactions, sex by itself has no main effect (χ2(1) = 3.126, p = 0.077) on IPH size; location, as expected, is tightly linked with IPH size and has a significant main effect (χ2(5) = 171.967, p < 0.0005); TC, binned by 10 mg/dL increments, is a significant predictor of IPH size (χ2(120) = 1828.766, p < 0.0005); age significantly affects IPH size (χ2(64) = 134.153, p < 0.0005); the components of TC (triglycerides, HDL and LDL) also each significantly affect IPH size (χ2(124) = 1999.491, p < 0.0005), χ2(75) = 301.292, p < 0.0005, χ2(135) = 506.425, p < 0.0005, respectively).

**Figure 2:**
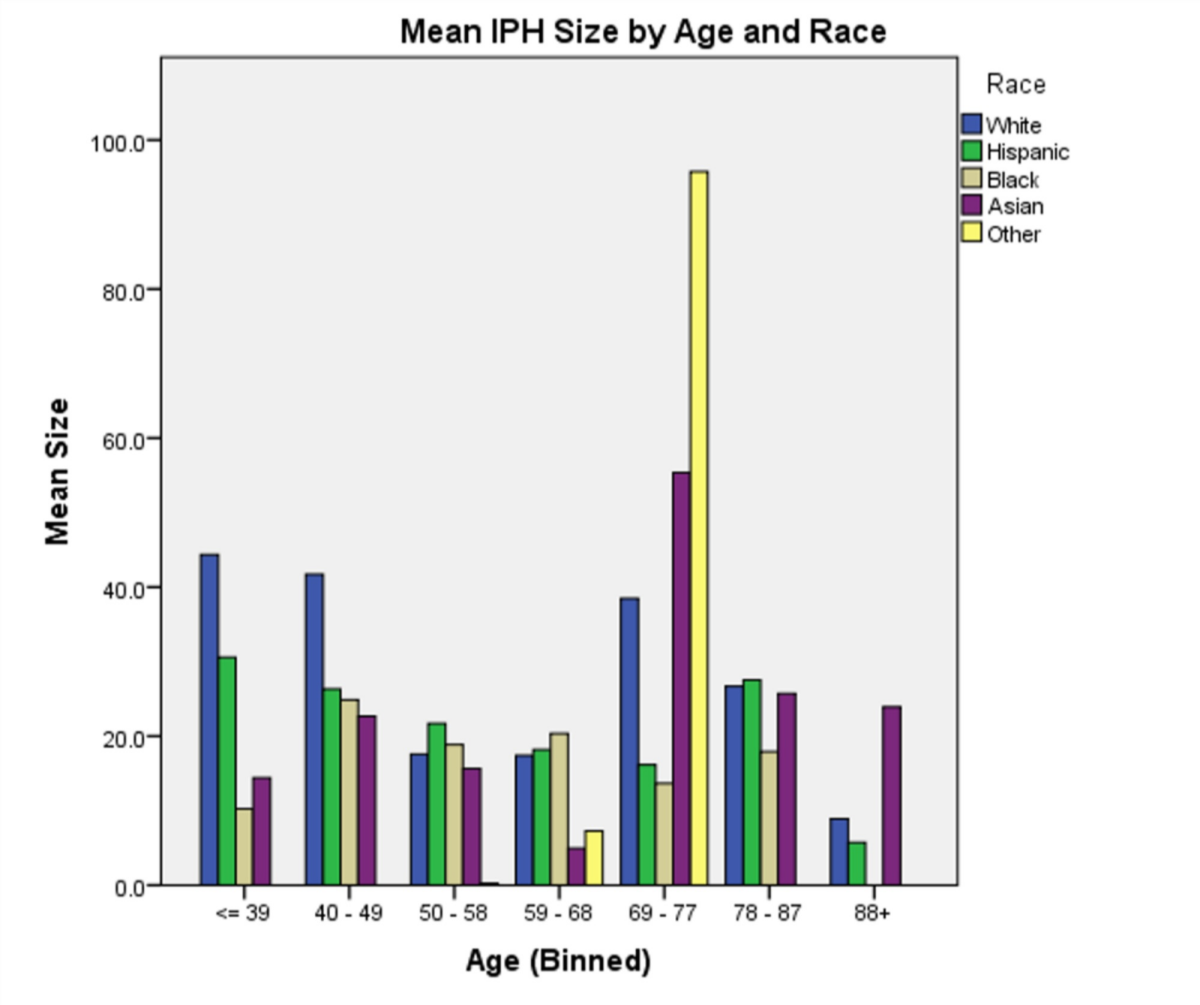
Mean intraparenchymal hemorrhage size by age and race Of note, there is generally a U-shaped distribution of intraparenchymal hemorrhage size with a nadir at 59 – 68 years except in black patients who nadir later at 69 – 77.

**Figure 3:**
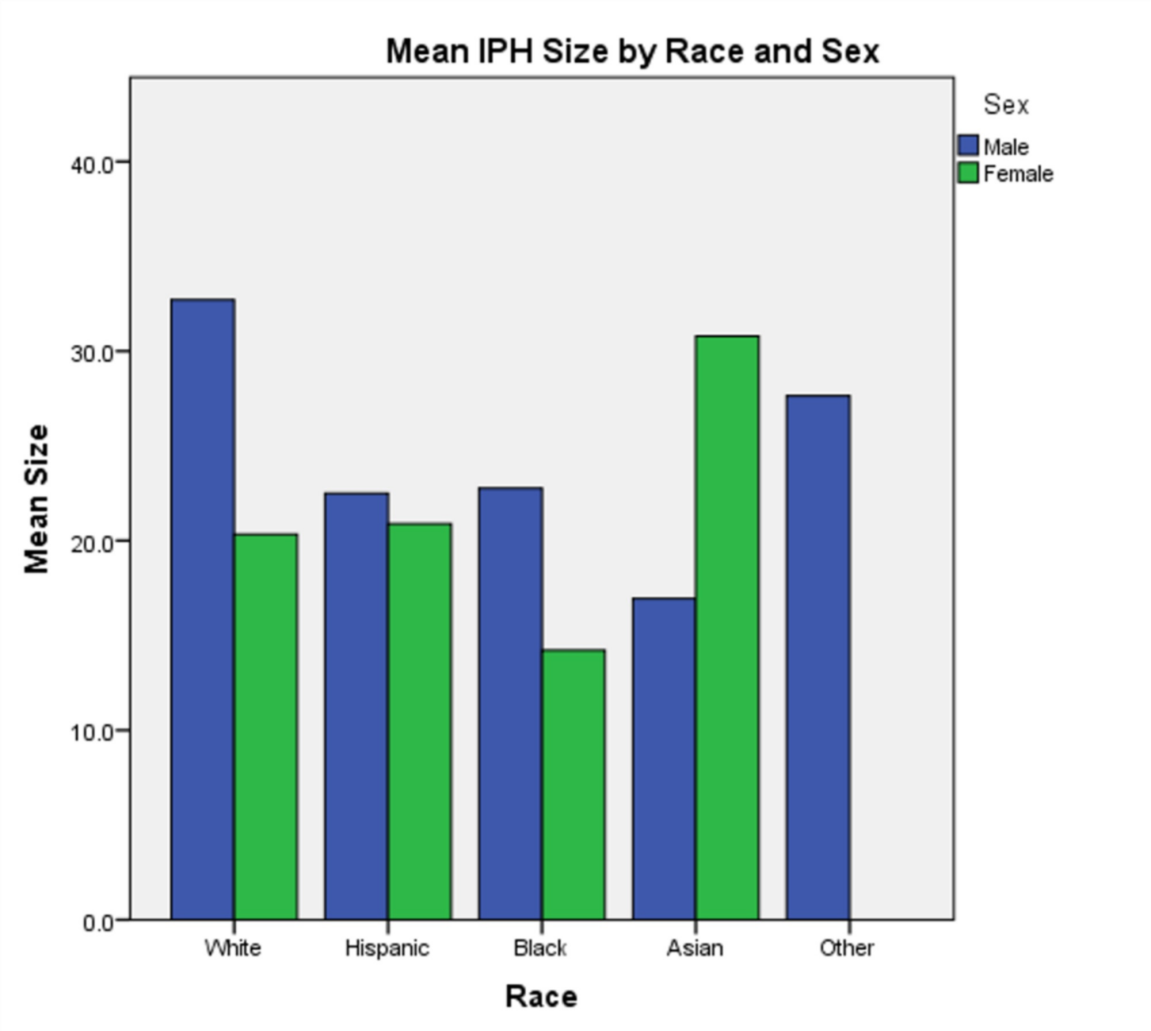
Mean intraparenchymal hemorrhage size by race and sex This figure demonstrates that black women and Asian men tended to have the smallest intraparenchymal hemorrhage sizes while white men and Asian women tended to have the largest.

**Figure 4:**
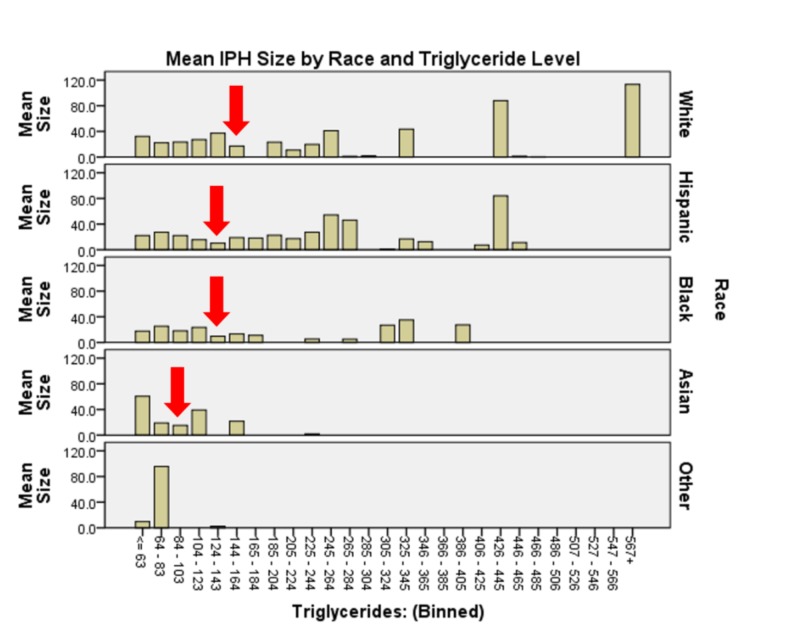
Intraparenchymal hemorrhage size by race and triglyceride level There appears to be a differential predilection for triglyceride ranges associated with smaller intraparenchymal hemorrhage sizes among the race categories with Asian patients presenting with smallest intraparenchymal hemorrhage sizes with triglyceride levels of 84 – 103 mg/dL, while hispanic and black patients have smallest intraparenchymal hemorrhage sizes with triglyceride levels of 124 – 143 mg/dL and white patients have smallest IPH sizes with triglyceride levels of 144 – 164 mg/dL.

In analyzing all two-way interactions, we see that all are significant except for race * TC. The interaction of age * race (χ2(70) = 305.272, p < 0.0005) shows that younger Asian, Hispanic and white patients tend to have larger hemorrhages than older patients in each respective group, then IPH sizes increase again after 69 years of age. This trend exists for black patients as well, however, for this group, the nadir occurs later (69 - 77) and increases again later (> 78) as compared to the white or Hispanic groups (Figure 2). The interaction of sex * race (χ2(3) = 11.209, p = 0.011) shows that black women and Asian men tended to have the smallest IPH size while white men and Asian women tended to have the largest (Figure 3). The interaction of race * triglycerides (χ2(9) = 25.515, p = 0.002) shows that minimum IPH sizes occurred at triglyceride levels of 144 - 164 mg/dL for white patients, 124 - 143 mg/dL for Hispanic and black patients, and 84-103 mg/dL for Asian patients (Figure 4).

**Figure 5:**
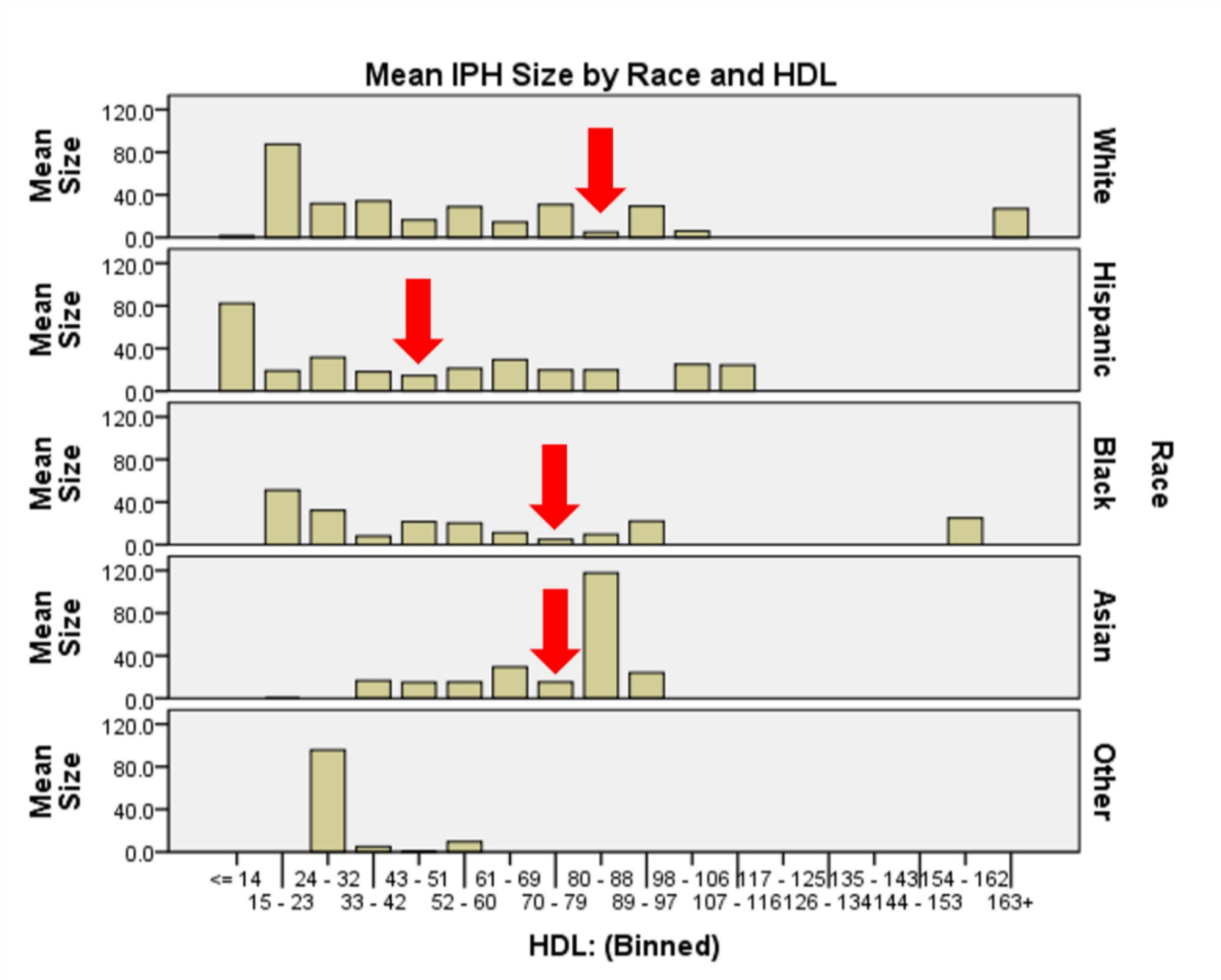
Graphical representation of serum high-density lipoprotein and race and the interaction effect on intraparenchymal hemorrhage size White patients required the highest serum high-density lipoprotein (80 – 88 mg/dL to minimize intraparenchymal hemorrhage size), Hispanic patients saw their minimum at 43 – 51 mg/dL, and black and Asian patients reached nadir intraparenchymal hemorrhage size at 70 – 79 mg/dL.

**Figure 6:**
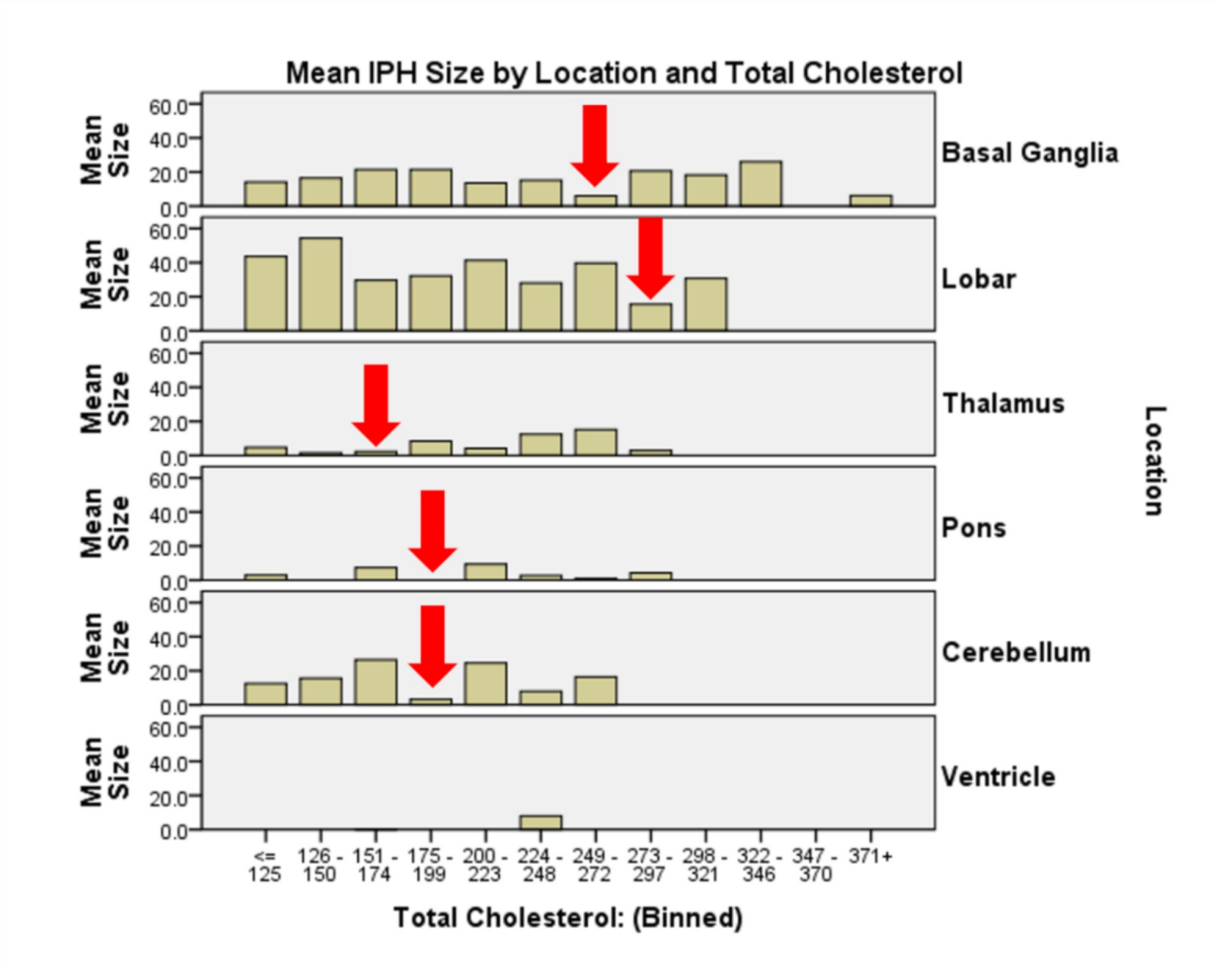
Graphical representation of a more granular view of serum cholesterol and intraparenchymal hemorrhage location interaction effect on intraparenchymal hemorrhage size Each location demonstrates different minimization ranges of total cholesterol for intraparenchymal hemorrhage size. Basal ganglia at 249 – 272 mg/dL, lobar hemorrhage at 273 – 297 mg/dL, thalamus 175 – 199, pons and cerebellum 175 – 199 mg/dL.

The interaction of race * HDL yields statistical significance on IPH size (χ2(5) = 2264.255, p<0.0005). Asian and black patients had the least IPH volume with HDL 70-79 mg/dL, while Hispanic patients had a minimum at 43 - 51 mg/dL. White patients required a higher HDL, 80-88 mg/dL to minimize IPH size (Figure 5). In the interaction of TC * age * sex *age (χ2(294) = 506863.86, p<0.0005), we see that on the whole, older and younger (< 49 and > 69) women have larger IPH volumes than age-matched men, while this trend reverses in the middle age group 50 - 68); cerebellar hemorrhages tended to happen with the highest frequency and largest volume in the 59-68 age demographic. Each location at each age bin demonstrates a different target range for TC resulting in minimal IPH size. Basal ganglia hemorrhages generally demonstrated the smallest IPH volumes with TC 249-272 mg/dL. Lobar hemorrhages, largest overall, demonstrated minimal IPH volume at TC 273-297 mg/dL. Thalamic hemorrhages had smallest IPH volumes with TC 151-174 mg/dL. Pontine and cerebellar hemorrhages demonstrated the smallest IPH volumes with TC 175-199 mg/dL (Figure 6). Table 2 represents a summary of the comparisons made and their p-values.

**Table 2:** Summary table of factorial statistical significance on intraparenchymal hemorrhage size Summary table of factorial statistical significance on intraparenchymal hemorrhage size. Using an alpha level of 0.05, significant effects are presented in bold. Of note, not all comparisons are represented in the table. Of all main effects, two-, three-, and four-way interactions, only race, sex, race*total cholesterol, sex*low-density lipoprotein*high-density lipoprotein, sex*low-density lipoprotein*location, and total cholesterol * low-density lipoprotein * high-density lipoprotein failed to reach significance at a p = 0.05 threshold.

Main Effects on IPH Size	p-value	Min IPH Size	Max IPH Size
Age	< 0.0005	50 – 58 & > 88	69 - 77
Race	0.518	Black	White
Sex	0.077	Female	Male
Location	< 0.0005	Pons	Lobar
Triglycerides	< 0.0005	205 – 224	245 - 284
Total Cholesterol	< 0.0005	188 - 196	128 – 136 & 223 - 231
LDL	< 0.0005	Multiple minima	Multiple maxima
HDL	< 0.0005	43 - 51	15 - 23
Two-way interactions on IPH Size			
Age * Race	< 0.0005	Asian 59 – 68	Asian 68 - 77
Race * Sex	0.011	Black Female	White Male
Race * Triglycerides	0.002	Black 144 – 164	White > 305
Race * HDL	< 0.0005	Black 70 – 79	Asian 80 - 88
Location * TC	< 0.0005	Thalamus 171 - 179	Lobar 249 - 257

## Discussion

The relationship between cholesterol and cardiovascular risk is long-studied. In particular, subtypes of cholesterol, HDL and LDL respectively have known specific end-organ effects. Namely, LDL delivers cholesterol to the end organs and has been associated with atheroma, while HDL binds and removes excess cholesterol from cells and tissues. Excess LDL is known to be a cardiovascular risk factor associated closely with heart disease and stroke. Another vascular lipid, triglyceride, has been shown to be inversely related to IPH. The overwhelming majority of literature aiming to determine optimal cholesterol goals are aimed at the prevention of cardiovascular disease or total disease burden and to our knowledge, this is the first study that has attempted to define a set of serum lipid goal ranges that may limit the extent of IPHs [1, 3-10, 17, 25-26].

This study used, as its predictive factors, patient age, sex, race, location of bleed, TCL, HDL and LDL levels, and triglyceride level. The results of the present study help to define specific goal ranges for each type of lipid, as well as for TC for the limitation of expansion of IPH in agreement with and in furtherance of existing literature [3, 7, 10, 15-16, 20-22]. This study did not, however, consider any cardiac issues or comorbidities in this analysis as this was outside the scope of this study. Our goal was to determine optimal cholesterol and component molecule ranges to minimize IPH size in hemorrhages that have already occurred. This, in concert with known, identified risk factors for the incidence of IPH may sharpen the prevention-treatment-recovery continuum for IPH patients and thereby improve patient outcomes in hemorrhagic stroke. See Table 3 for a summary of known risk factors for IPH.

**Table 3:** Risk factors for intraparenchymal hemorrhage Adapted from An SJ, Kim TJ, Yoon BW; emphasis added [5].

Modifiable risk factors
Hypertension
Current smoking
Excessive alcohol consumption
Decreased low-density lipoprotein
Low triglycerides
Anticoagulation
Use of antiplatelet agents
Sympathomimetic drugs
Non-modifiable risk factors
Old age
Male sex
Asian ethnicity
Cerebral amyloid angiopathy
Cerebral microbleeds
Chronic kidney disease
Other factors
Multi-parity
Poor working conditions (blue-collar occupation, longer working time)

Considering single main effects, this study did not show a statistical main effect of sex on bleed size (p=0.077). While it may appear, at first, that this disagrees with existing literature, it is important to note that existing literature on sex differences in IPH has considered the only occurrence of IPH by sex, not the size of IPH by sex. Age demonstrated a U-shaped distribution with middle-age (59-68 years) subtending the smaller hemorrhage volumes. As would be expected, hemorrhage size was significantly predicted by hemorrhage location with smaller brain areas subtending smaller IPH (p<0.0005). TC had a significant main effect on bleed size (p < 0.0005) with the smallest hemorrhages, across all hemorrhage locations and patient genders occurring at TCLs 181-196 mg/dL. HDL, a component of TC, has long been known as the “good cholesterol” and this moniker holds mainly true in this study with smallest bleed sizes occurring at an HDL level 43-51 mg/dL, a level that is consistent with current recommendations.

As is expected, women have a generally higher baseline HDL, all other conditions being equal, than do men. This study demonstrates that women, additionally, have a higher HDL level than men at minimal IPH size in each bleed location. Men in this study, if we include all data, without correction for outliers, had minimum bleed sizes in HDL range 98 - 106 mg/dL while women had minimum bleed size at 43-51 mg/dL. While this study does not address the biochemistry and physiology of this difference, previous literature suggests that HDL levels are increased by serum estrogen and, the protective effects of HDL may be potentiated by circulating estrogen. It is interesting to note that despite the amount of attention that LDL garners in the cardiovascular literature and clinical recommendations and our study demonstrated a statistically significant effect of LDL on IPH volume (p<0.0005), there was no clearly identifiable directionality of the trend. This is consistent with the metadata reported by Goldstein (2009) and highlights how complex the interrelationships between serum lipids, cardiovascular disease, and cerebrovascular disease are [14]. There may be many reasons for this observation and may in part be due to the less well reported effects of LDL, namely transport of fat-soluble vitamins and antioxidants to cells. Perhaps these antioxidant and nutritive effects preferentially protect neural and neurovascular structures and this protective effect balances the potentially harmful effects of LDL. Of course, this is best left for specific evaluation in future work. There appears to be a differential predilection for triglyceride ranges associated with smaller IPH sizes among the race categories with Asian patients presenting with smallest IPH sizes with triglyceride levels of 84-103 mg/dL, while Hispanic and black patients have smallest IPH sizes with triglyceride levels of 124-143 mg/dL and white patients have smallest IPH sizes with triglyceride levels of 144-164 mg/dL. Of note, in the 386-405 mg/dL range of serum triglycerides, there is a dramatic increase in bleed size. This is consistent with the Atherosclerosis Risk in Communities (ARIC) study and the Cardiovascular Health Study (CHS) reports that low triglyceride levels were associated with increased risk of IPH and the work of Do that confirms that excessively high triglyceride levels contribute to atherosclerosis and atheroembolic events [3, 14, 27]. Along those lines, it is important to note that this study considers only IPH stroke and that risk factors and effect sizes may well be different in ischemic stroke.

When reintroducing other individual factors to consider two-way interactions of predictor variables on IPH size, other interesting effects arise. The interaction of sex * location (p<0.0005) demonstrates that in the basal ganglia, cortical lobes, and cerebellum, IPH in men were larger, while in the thalamus and pons, IPH were larger in women. This perhaps indicates some developmental predilection to particular vascular patterning or other anatomic or physiologic patterning that differentially subtends these areas between men and women. The interactions between sex and HDL and location and HDL are likewise statistically significant (sex * LDL p<0.0005, sex * HDL p=0.024). As noted previously, the maximally protective HDL level for men is 98-106 mg/dL while for women it is 43 - 51 mg/dL. As mentioned above, the reason for increased female HDL level is likely to be related to circulating estrogen levels and a potentiating effect that estrogen may have on vasoprotective HDL.

Triglycerides, again, show significant two-way interactions with both sex (p<0.0005) and location (p<0.0005). The optimal range of triglycerides producing minimum bleed sizes occurred at 205 - 224 mg/dL. These levels coincide with mildly elevated levels in clinical practice. This range producing minimum bleed size was conserved between the sexes and among all locations. This effect is more nuanced when considered with race with statistically significant minimum IPH size occurring at 144-164 mg/dL for white patients, 124-143 mg/dL for Hispanic and black patients, and 84-103 mg/dL for Asian patients (Figure 4). It is important to note, however, that men, on average, had higher triglyceride levels than women and that men with triglyceride levels > 400 mg/dL had vastly larger hemorrhage sizes. In this study, very few women had triglycerides that were so elevated, however, those few that did present with much smaller bleeds than did their male counterparts. Of uncertain clinical value, though important to note, bleeds of patients with “high triglycerides” tended to occur in the basal ganglia with extremely large and catastrophic hemorrhage at triglyceride levels > 400 mg/dL occurring almost exclusively as lobar hemorrhage.

Now considering three-way interactions, sex, location, and TC were statistically significant (p<0.0005). This implies that the optimal range noted previously, 188-196 mg/dL of TC has a strong enough effect that it transcends gender and hemorrhage location. This is to say that across all genders and all hemorrhage locations, this range of TC was observed to yield minimum hemorrhage sizes. The three-way interaction of sex, location, and HDL warrants some further discussion. Above, if the sample is considered with obvious outliers, it was noted that overall, HDL 43-51 mg/dL was maximally protective against IPH expansion. This, however, means, that with optimal triglyceride range, 205-224 mg/dL, using the formula TC = TG/5 + HDL + LDL, to meet the optimal TC range of 188 - 196 mg/dL, a patient should have a target LDL range of 100.2-104. While this is, of course, impractical, it is important to recall that LDL is a carrier molecule for fat soluble vitamins and antioxidants and clinical application of this postulation should be deferred until this relationship is better defined.

## Conclusions

This study suggests that it is possible that there may be optimal serum lipid ranges for TC and component molecules in minimization of IPH sizes through all genders and hemorrhage locations. This study proposes there may be an optimal neuroprotective range that falls within the generally accepted cholesterol guidelines of TC with additional minimum ranges identified for HDL and triglyceride levels, nuanced by sex and race. Although LDL did meet statistical significance, there was no identifiable trend that would suggest a particular target for LDL in the IPH patient. A granular approach that involves the physiologic and biochemical pathways for each of these molecules in circulation, as well as neuronal tissue development and stabilization, is needed to evaluate modification of the component molecules of cholesterol as a clinical paradigm. Future work in this area should include collaboration with cardiology investigators to determine ideal ranges that simultaneously address both cardio- and neuroprotection as well as a prospective study to validate the applicability of these findings in patient care.
